# Migrants’ health and persisting barriers to accessing health-care systems

**DOI:** 10.1016/j.eclinm.2022.101321

**Published:** 2022-02-24

Migration is a global reality in the current world, with the more prominent dialogue often related to migration to high-income countries. Movement into new countries present multiple challenges and one of them is for migrants to be able to access and integrate into the health-care system of host countries. According to the European Charter of Fundamental Rights, all individuals have the fundamental right to health care, with the minimum requirement to be able to access emergency care. But is this right a reality for most migrants?

There is no formal internationally recognised definition of a migrant, but according to the International Organisation for Migration, a migrant is “anyone who has moved either across or within state borders away from their usual residence”. In Europe, on Jan 1, 2020, 5.1% (23 million) of the EU total population were non-EU citizens and 8.3% (13.1 million) of all EU inhabitants were not born in EU, according to data from Eurostat. However, although often seen as a single group, migrants are extremely heterogeneous in terms of provenience, reasons to move from their country of origin, burden of disease, and lifestyle health-related risks. Migrants can also be a very heterogeneous group in terms of their rights and entitlements, with undocumented migrants, such as asylum-seekers and refugees, facing more difficulties in accessing health-care systems of the host country than documented migrants.

Although the basic entitlement to health care is shared across different European countries for migrants with a regular immigration status, the situation is more variable in relation to undocumented migrants and represents a huge barrier to accessing health care. For example, 2016 data from the European Union Agency for Fundamental Rights suggest that among 28 countries from the EU, eight countries allow access to emergency, primary, and secondary care to undocumented migrants only upon payment, six countries allow undocumented migrants to access the same services entirely for free, whereas the remaining 14 countries present a mixed situation, where emergency care can be accessed for free, but primary and secondary care cannot.

However, even when legal accessibility is available, inequalities still exist in accessing health care due to more informal barriers, such as a lack of knowledge of the new health systems or of the language of the host country. Evidence of migrant's access to health care is also very fragmented, especially in relation to undocumented migrants. A recent meta-analysis analysing documented regular migrants’ access to health care in Europe confirmed that uneven use of health care also persists in this group. Studies from nine countries, namely Austria, Cyprus, France, Germany, Greece, Italy, Malta, Spain, and Sweden, showed an increased use of emergency room or acute care provisions by migrants compared to native people and a lower access to routine primary care services. In agreement with these data, the 2018 WHO report on health of refugees and migrants in the European Region noted that compared with non-migrants, migrant women face poorer pregnancy and birth outcomes with a higher incidence of caesarean sections and complications, suggesting a lower access to family planning and prenatal checks. The WHO report also highlights how migrants’ children are more prone to have diet-related issues compared with native children. The data available clearly signals the presence of barriers to migrants’ use of routine services, with a higher risk of negative health outcomes.

The WHO report calls for the development of more migrant-friendly health systems and also for more data focused on the specific individual health needs. More attention should be paid to the country of origin, health burden, and health-related risks to be able to identify areas for action plans and tailor specific inclusive policies. There is a need to strengthen policies in relation to undocumented migrants, with the aim to make health care accessible across all European countries. Furthermore, we need to build action plans and frameworks based on data to be able to overcome barriers. For health systems to be more migrant-friendly, we would need to eliminate cultural barriers with, for example, the possibility to have an interpreter when needed and more support for newcomers with registering to the health systems and knowing their rights. The health-care workforce should also be trained to be more aware of the differences in burden of disease and health risks of migrants compared with native people to be able to set up a plan of preventive visits and to enable regular access to care. Early diagnosis and treatment will bring benefits as they are associated with less treatment costs in the long term and less access to emergency care services.

The crucial work of different charities is pivotal to help support migrants in need. However, guaranteeing access to health care as a basic human right is a governmental task and therefore a top-down inclusive framework to make health-care systems more migrant-sensitive should be developed by governments globally. In this context, the sometimes negative narrative on migrants should be changed towards highlighting how migrants contribute, or could contribute, actively to society, and to their positive impact on the economy of the host country. This is also important in the context of the declining birth rates registered in many European countries, and the need for an international workforce in many work sectors. Inclusion and integration at all levels will be required as pillars for future governments’ strategies to finally reach universal health coverage and “health for all”.

